# Intrinsic Solubility of Ionizable Compounds from p*K*_a_ Shift

**DOI:** 10.1021/acsomega.3c04071

**Published:** 2023-11-13

**Authors:** Joku̅bas Preikša, Vilma Petrikaitė, Vytautas Petrauskas, Daumantas Matulis

**Affiliations:** † Department of Molecular Compound Physics, Center for Physical Sciences and Technology, Savanoriu Ave. 231, Vilnius, LT-02300, Lithuania; ‡ Department of Biothermodynamics and Drug Design, Institute of Biotechnology, Life Sciences Center, Vilnius University, Saulėtekio 7, Vilnius, LT-10257, Lithuania; ¶ Laboratory of Drug Targets Histopathology, Institute of Cardiology, Lithuanian University of Health Sciences, Sukileliu pr. 13, Kaunas, LT-50162, Lithuania

## Abstract

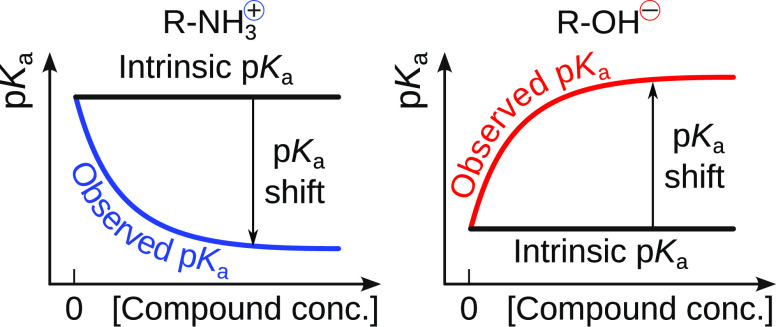

Aqueous solubility of pharmaceutical substances plays an important role in small molecule drug discovery and development, with ionizable groups often employed to enhance solubility. Drug candidate compounds often contain ionizable groups to increase their solubility. Recognizing that the electrostatically charged form of the compound is much more soluble than the uncharged form, this work proposes a model to explore the relationship between the p*K*_a_ shift of the ionizable group and dissolution equilibria. The model considers three forms of a compound: dissolved-charged, dissolved-uncharged, and aggregated-uncharged. It analyzes two linked equilibria: the protonation of the ionizable group and the dissolution–aggregation of the uncharged form, with the observed p*K*_a_ shift depending on the total concentration of the compound. The active concentration of the aggregates determines this shift. The model was explored through the determination of the p*K*_a_ shift and intrinsic solubility of specific compounds, such as ICPD47, a high-affinity inhibitor of the Hsp90 chaperone protein and anticancer target, as well as benzoic acid and benzydamine. The model holds the potential for a more nuanced understanding of intrinsic solubility and may lead to advancements in drug discovery and development.

## Introduction

Aqueous solubility of any compound developed as a drug is an important property affecting its ADME (absorption, distribution, metabolism, and excretion) properties, and together with membrane permeability, it directly affects oral bioavailability.^1^ Many functionally active compounds failed to become drugs because of their poor physicochemical properties.^2^ Researchers examine the physicochemical characteristics of compounds in the early stage of drug development to initiate the bioavailability enhancement of poorly soluble compounds.^3,4^

Compounds that contain ionizable groups exhibit the dependence of solubility on pH. For this reason, p*K*_a_-solubility profiling has become a common practice in drug research and industry.^5−7^ For example, Bergström et al.^8^ observed differences in solubility between the charged and uncharged drug species. The electrostatically charged compound was significantly more soluble than its uncharged form. This pH-dependent solubility phenomenon must be considered when determining the compound’s intrinsic solubility and studying its binding to target proteins.

Intrinsic solubility is defined as the saturating concentration of the compound’s electrostatically neutral (or nonionized) form. Various *in silico* models were developed to predict the intrinsic solubility.^9,10^ However, the models are usually based on data-driven machine learning techniques and require high-quality intrinsic solubility data. In some cases, using empirical data from parallel experiments can be even more challenging than the experimental determination of intrinsic solubility.^10−16^ With increased computational power, it becomes more convenient to derive high-quality descriptors based on *ab initio* calculations to minimize the data required for statistical training models.^17^

The electrostatically neutral form of a compound may aggregate and change the observed equilibria. For example, Kawakami et al.^18^ demonstrated that the amount of solid in the system affects the observed solubility. In another study, Mohammadi et al.^19^ showed that the solubility of ionizable compounds could be marginally affected by the introduction of excess solid form. Despite some hypothetical explanations involving dimer formation or partitioning (adsorption) of the compound onto the excess solid in the suspensions,^20^ these processes still need a better understanding. As a result, it is advised to keep the excess of the added compound 2–4 fold over equilibrium solubility. However, such concentrations are sometimes challenging to achieve, especially for compounds with low aqueous solubility.^21^

Here we apply a model which correlates the p*K*_a_ shift of a compound ionizable group with its observed aqueous solubility and enables determination of the intrinsic solubility of benzoic acid, benzydamine,^22,23^ and the compound ICPD47, an inhibitor of Hsp90 human chaperone.^24−26^ The compound is similar to a series of Hsp90 inhibitors in anticancer clinical trials and was among lead compounds for further development.^27^ While attempting to determine the intrinsic thermodynamics of ICPD47 compound binding to Hsp90 it became clear that the p*K*_a_ shift is also linked to compound aggregation. It was difficult to determine the p*K*_a_ of the compound, and the p*K*_a_ depended on the concentration used in the experiment. Thus, we pursued the issue and built the model to account for the observed p*K*_a_ shift. Furthermore, to demonstrate the general applicability of the model, we searched for literature examples and showed that the model is also applicable to benzoic acid and benzydamine. These compounds are structurally unrelated to ICPD47, and they bear the negatively charged carboxylic group and positively charged amino group, respectively. As predicted by the model, the shift occurred in opposite directions for the oppositely charged compounds, thus strongly supporting the general applicability of the model.

## Materials and Methods

### Chemicals

The compound ICPD47 was synthesized at the Department of Biothermodynamics and Drug Design (Vilnius University, Lithuania) as previously described.^24^ The compound was chemically stable in acid and base (pH 2.0 to 12.0). The stock solution of the compound was prepared in DMSO and stored in the dark at (4 ± 2) °C for less than one month. The stock solutions of all chemicals were prepared in degassed and boiled milli-Q water to prevent CO_2_ interference with pH titrations. The stock solutions of 1 M hydrochloric and nitric acids were standardized volumetrically using 1 M Tris and NaOH solutions to ensure the precision of each concentration is within ±2%. The stock solution of 1 M NaOH (Ph. Eur. grade) was bought from Sigma-Aldrich.

### Potentiometric Titration at the Concentrations Exceeding Solubility

The compound ICPD47 is sparingly soluble at near-neutral pH. Solubility is approximately equal to or below 100 μM. The solutions exceeding the solubility limit (0.25, 0.5, and 1.0 mM) were prepared by adding 1.5 equiv of NaOH (0.375 0.75, and 1.5 mM, respectively; 0.5 equiv excess), thus shifting the compound to its highly soluble deprotonated negatively charged form. The deprotonated solutions were titrated with 20 times more concentrated HCl (2.5 mM; 5.0 mM; and 10 mM) containing the same concentration of DMSO as in the analyte solution (2.0% in all solutions). Repeating potentiometric titration at several DMSO concentrations (0.5%; 1.0%; and 2.0%) did not affect the results within the error of the measurements.

The pH titration was performed with a 250 μL syringe that was filled with the titrant (acid), and the needle was placed into the sample cell filled with 2.5 mL of analyzed solution. A pH microelectrode was also inserted into the same cell. The titrant was added in 25 aliquots of 10 μL at a fixed rate of one injection per minute. The total volume of added titrant was 250 μL. The analyzed solution was mixed after each injection using a pipet. The pH was measured potentiometrically using a Mettler Toledo MP220 pH meter with a combined glass InLab Microelectrode. The pH meter was calibrated before and checked after each titration using the standard pH solutions. The pH measurements were recorded only when the pH measurement instability was less than 0.01 min^–1^. The temperature was kept constant at 25 °C.

### Spectrophotometric Determination of the p*K*_a_ Shift

The ICPD47 compound solutions were prepared in the universal buffer of different pH values from 4.5 to 10.5 at every 0.5 pH unit. The total added compound concentrations were 0.025, 0.05, 0.1, 0.2, 0.5, 1, and 2 mM, below and above the solubility limit. To prevent light scattering, each analyte solution was centrifuged at 13500 rpm for 1 min. The absorbance was measured with an Agilent 8453 UV–vis spectrophotometer using the same 2.5 mL cuvette as for potentiometric titration. The wavelength range of the scan was 200 to 600 nm. The temperature was controlled at 25 °C. The universal buffer solutions contained 50 mM NaCl, 50 mM sodium acetate, 25 mM sodium tetraborate, and 50 mM sodium phosphate, with the pH adjusted every 0.5 unit.

### Literature Data

As mentioned above, to support our model, previously published data were collected.^22,23^ Since raw data formats were unavailable, we used WebPlotDigitizer to extract data from Bjerrum plots.^28,29^ Additionally, we used the equation for bound hydrogen ions per substance molecule to reverse engineer equivalent titrant added during the titration.^30^

## Results

### The p*K*_a_ Dependence on the Amount of Nondissolved Compound

We attempted to determine the p*K*_a_ of compound ICPD47 and noticed that the resultant value of the p*K*_a_ strongly depends on the concentration used for the titration. When we made a determination at the concentrations exceeding the apparent solubility, we observed the p*K*_a_ shift toward higher pH. The same result was observed by two independent techniques, both the spectrophotometric and potentiometric measurements. To determine the p*K*_a_ of the compound we applied the model of p*K*_a_ shift correlation with the solubility, similar to our previously derived model for amine protonation-aggregation for the determination of the hydrophobic effect.^31−33^

**Figure 1:**
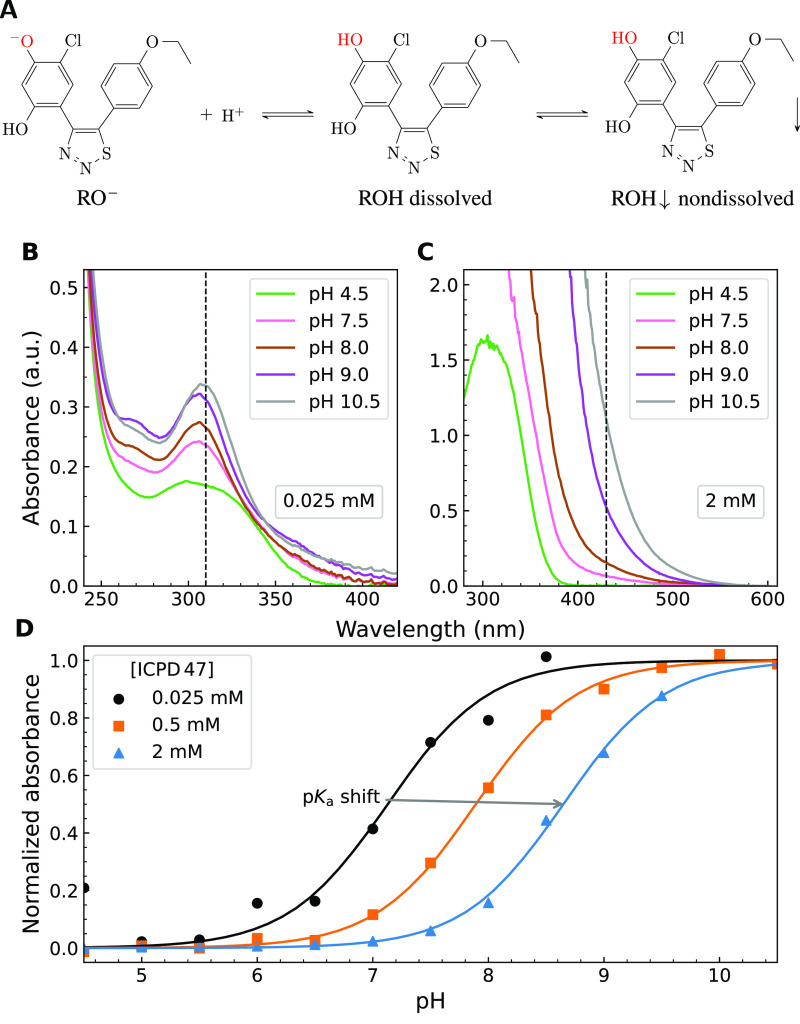
Three forms of ionization and dissolution of the compound ICPD47: highly soluble and negatively charged deprotonated form, the electrostatically neutral dissolved form, and the electrostatically neutral aggregated (nondissolved) form (A). Absorbance spectra of the ICPD47 compound at 0.025 mM concentration (B). Absorbance spectra of ICPD47 compound at 2 mM concentration (C). The vertical dashed line shows the arbitrary wavelength chosen to calculate the p*K*_a_. (D) The normalized absorbances obtained from spectra as in panels B and C as a function of pH showing the shift of the p*K*_a_.

The compound ICPD47 exhibits a limited micromolar solubility and strongly depends on pH. Upon deprotonation of the phenolic hydroxy group, the solubility of the deprotonated form is greater than that of the protonated form (Figure 1A). The protonated electrostatically neutral form of the compound may exist in two forms—dissolved or nondissolved (aggregated). The deprotonated negatively charged form is assumed to exist exclusively in the dissolved form. However, it may also exist in a nondissolved form, but we excluded it from the model since the ionized form is likely 100 to 1000 times more soluble than the nonionized form. At higher (alkaline) pH, the compound exists mainly in a highly soluble deprotonated negatively charged form. At lower (acidic) pH, the compound should exist in protonated uncharged forms—dissolved and aggregated.

There are two hydroxy groups on the Hsp90 compound that can become deprotonated. According to the p*K*_a_ measurements of similar compounds such as chloro resorcinol, the first hydroxy group to become deprotonated is most likely at the ortho position to the Cl atom. However, the exact position does not alter the applicability of the model.

The spectrophotometric determination of the compound p*K*_a_ exhibited the shift of the observed p*K*_a_ (Figure 1B-D) at large added compound concentrations. The intrinsic solubility of the uncharged form of the ICPD47 was determined to be approximately 75 μM. The total added concentrations of 0.5 and 2 mM were much greater than the intrinsic solubility. However, the p*K*_a_ shift was clearly observed at every added compound concentration. The spectra did not change with time (within 5 to 60 min intervals), thus indicating that the dissolution equilibrium has been achieved and the observations are not due to kinetic or temporary supersaturation reasons.

**Figure 2:**
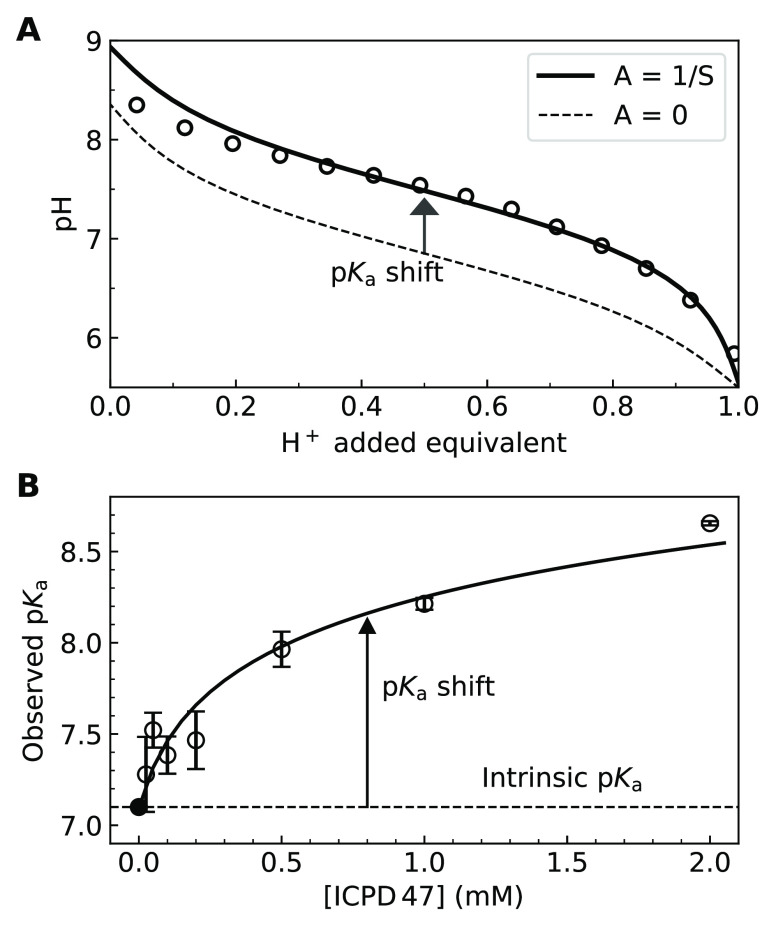
Potentiometric titration of ICPD47 at a 0.25 mM concentration (panel A, open circles). The data were fit by assuming the A parameter in the model to be zero (no shift at infinitely low concentration, dashed line) and with the shift (solid line, 0.25 mM). The observed p*K*_a_ shifted toward higher pH upon the titration at higher compound concentrations. Spectrophotometric observed p*K*_a_s as a function of the total added compound ICPD47 concentration (B). The intrinsic p*K*_a_ was concentration-independent, while the observed p*K*_a_ values shifted with an increase in ICPD47 concentration as predicted by the model (solid line).

In the potentiometric titration, we also observed similar results. If the compound was prepared by deprotonating it with 1.5 equiv of sodium hydroxide and then titrated back with the strong acid, there was a shift of the observed p*K*_a_ (Figure 2 A). This shift is due to partial aggregation of the protonated electrostatically neutral form of the compound. At higher added concentrations, the shift is larger because there is a stronger pull toward precipitation.

Figure 2B shows the shift of the observed p*K*_a_s as a function of the total added ICPD47 concentration. The compound intrinsic solubility was then determined by applying the model explained below to be approximately equal to 75 μM. Protonation at higher concentrations led to the precipitation of the electrostatically neutral form. The apparent observed p*K*_a_ of the compound shifted to the alkaline side because of the pull by the aggregated form, but the true intrinsic p*K*_a_ did not depend on the concentration of the added compound.

### The Model of Linked Ionization and Dissolution

According to the model, any protonizable compound exists in three forms, the charged-dissolved form, the uncharged-dissolved form, and the uncharged-nondissolved (aggregated) form. We use the concept of ‘the active concentration of an aggregate (precipitate)’. This concentration is mathematically equal to the amount of aggregate divided by volume of solution. This aggregate remains undissolved and is at an equilibrium (thermodynamic) concentration. The uncharged form of the ICPD47 compound exists in two forms, the dissolved form [ROH] and the aggregated form [ROH*↓*]. Furthermore, the total concentration of the ICPD47 compound, *C*, is equal to the sum of all three forms of the compound and includes the deprotonated concentration [RO^–^]:
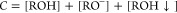
1Here we assume that the deprotonated form is highly soluble and there is no need to consider the aggregated form of the negatively charged deprotonated compound. The following three reactions occur upon titrating the compound with the hydrochloric acid:
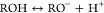
2
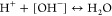
3
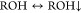
4The acid-dissociation (protonation) constant, *K*_a_, is defined as
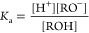
5The total concentration *C* (eq 1) is equal to the concentration of sodium ions, [Na^+^] because at the beginning of the titration, the compound existed entirely as an anion and the sodium cation was needed to adhere to the principle of electrostatic neutrality (similar derivations of various titration curves are described in ref (34)).
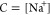
6An aggregation constant, *A*, could be used to describe the dissolution equilibrium. *A* is inversely proportional to the intrinsic solubility, *S*, and is expressed and rearranged to
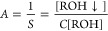
7
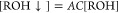
8The dissociation constant of water is
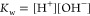
9The charge balance must be preserved during the titration:
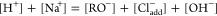
10The added concentration of Cl^–^ is equal to the added concentration of H^+^ because these ions are added together:
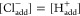
11We can now combine seven equations eqs 1 and 5–11 to calculate the seven species in the equilibria, [ROH], [RO^–^], [ROH*↓*], [Cl−], [H_add_^+^], H^+^, and [OH^–^], in the terms of the parameters *K*_a_, *K*_w_, *A*, and *C*, and thereby predict the titration curves. The resultant equation
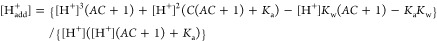
12cannot be expressed as a function pH = *f*([H_add_^+^]), but may be solved numerically. The predicted titration curve and its shift dependent on the concentration of aggregated form are shown in Figure 2 A.

The concentrations of each form of the compound may be calculated as a function of solubility (aggregation constant *A*), total added concentration (*C*) and pH:
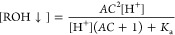
13
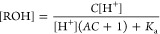
14
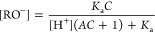
15

**Figure 3:**
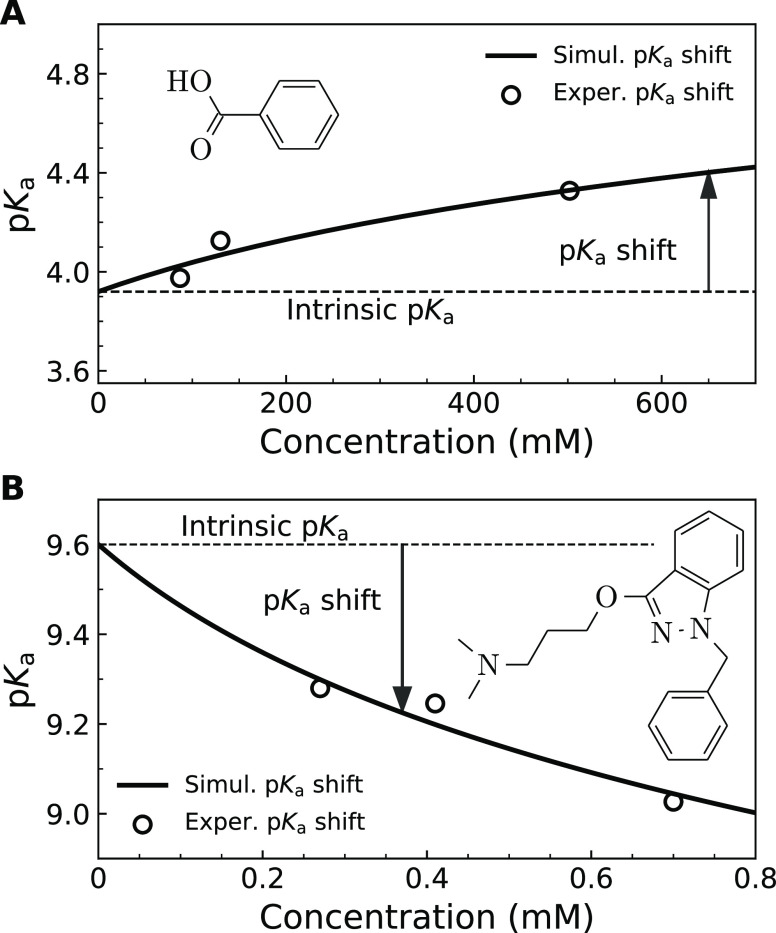
Observed p*K*_a_ shift of benzoic acid (A) and benzydamine (B) titrations as a function of their total concentration when performing the titration. Application of the model showed that the intrinsic p*K*_a_s were equal to 3.92 and 9.60, respectively. The intrinsic solubilities were equal to 0.32 and 0.27 mM, respectively. The data points (open circles) were taken from ref (5), and the model (solid lines) well accounted for the p*K*_a_ shifts. Note that the benzoic acid p*K*_a_ shift is directed toward alkaline pH because the benzoic acid aggregate is protonated, while the benzydamine p*K*_a_ shift is directed toward acidic pH because the benzydamine aggregate is deprotonated.

We analyzed several literature examples where ionizable compound titration curves have been published, and the concentration-dependent p*K*_a_ shift was observed. The book by Avdeev^5^ contained the p*K*_a_ shift of benzoic acid and benzydamine. Benzoic acid may dissociate to negatively charged benzoate, and therefore the p*K*_a_ shift occurs toward the alkaline pH direction. However, benzydamine is an amine that becomes positively charged and thus more soluble upon protonation and the p*K*_a_ shifts downward. Both shifts are well accounted for by our model (Figure 3) solid lines predicting an increased p*K*_a_ shift at higher added compound concentrations. The intrinsic solubilities of benzoic acid and benzydamine were found to be equal to 0.32 M and 0.27 mM, and the intrinsic p*K*_a_ were equal to 3.92 and 9.60, respectively. Such p*K*_a_ values would be observed only at infinite dilution.

## Discussion

It is usually assumed in physical chemistry that the activity of the nondissolved (aggregated, precipitated) form is equal to unity and that a compound’s aggregated (nondissolved) form does not participate in the thermodynamic equilibrium of dissolution. Here we see that the observed p*K*_a_ of ICPD47, benzoic acid, and benzydamine deprotonation depends on the amount of aggregated form. Therefore, we use the concept of ‘the active concentration of an aggregate (precipitate)’. This concentration is mathematically equal to the amount of aggregate divided per volume of solution. This concentration of material that remains undissolved is an equilibrium (thermodynamic) property. According to our model, the uncharged form of the compound may exist in two forms, the dissolved form and the aggregated form. Furthermore, the total concentration, *C*, of all three forms of the compound includes the charged concentration.

It is often considered that an entire amount of any compound is fully dissolved if its concentration is below the solubility value. It is also considered that if surplus of a compound is added above the solubility limit, all extra added compound should remain in a nondissolved (aggregated) form, presumably precipitated on the bottom of solution. The same should be valid if there are two ionization forms of the compound: the ionized form should be fully dissolved, while the neutral form should be partially dissolved and partially aggregated.

Contrary to these conventional assumptions, our model states that all forms of the compound may coexist at any pH and at essentially any added concentrations. All forms are in equilibrium, and their relative concentrations depend on the total added concentration and pH. At conditions where half of the un-ionized compound is dissolved, half of the compound should remain aggregated. Most importantly, according to our model, the aggregated form will actively participate in the dissolution equilibrium and any other thermodynamic equilibria, e.g., the compound binding reaction to a protein. Saturation of the protein with the ligand could be higher if there were a higher amount of aggregates present in the system.

Another example of the participation of the aggregated form in the linked equilibria could be the shift of the observed p*K*_a_ as discussed in this manuscript. The shift would not depend on the amount of added precipitate if it did not fully participate in the thermodynamic equilibrium of dissolution in water. This observation cannot be assigned to the kinetic or supersaturation reasons because the spectrophotometric measurements remained unchanged for an extended period, indicating that equilibrium has been achieved.

This model of compound dissolution is similar to our previously described model of the aliphatic amine-linked protonation and aggregation.^31−33^ Aliphatic amines also exist in three forms: the protonated (positively charged amine, highly soluble) form, the deprotonated-dissolved form, and the deprotonated-aggregated form. Dissolution of the deprotonated (uncharged) amine was highly dependent on the aliphatic chain length. Each methylene group decreased the solubility by approximately 4-fold. The observed p*K*_a_ of the amino group depended on the aliphatic chain length, total added concentration, and pH. However, the intrinsic p*K*_a_ was independent of these variables. In these terms, the models are similar. Some inconsistency between the observed and theoretically modeled curves was due to the cooperative Scatchard-Black effect,^35^ which explains how the precipitation of the compound in electrostatically neutral form either pulls the protons out of solution or gives them to the solution to make the compound electrostatically neutral upon precipitation. The resultant increase or decrease of pH depends on whether the compound can become negatively or positively charged.

The *intrinsic* binding constant for a thermodynamically well-defined system should not depend on the concentration of the added reactants. However, the *observed* binding constant may depend on the total added concentration if an aggregation reaction is involved. This dependence was also observed when the positively charged aliphatic amines interacted with the negatively charged aliphatic sulfonates.^36^ Their complexes had low solubility and aggregated. Thus, at higher concentrations, the observed binding constant was larger by the same proportion.

The observed p*K*_a_s and solubilities of various pharmaceutically important molecules are usually experimentally measured at low concentrations. However, they still may be influenced by the linked aggregation and ionization equilibria. Pharma Algorithms software “ADME/Tox boxes” (4.9 version) predicted the p*K*_a_ of ICPD47 to be 7.4 ± 0.8. The predicted solubility *S*_w_ was equal to 0.058 mg mL^–1^ (166 μM). Our model estimated the p*K*_a_ to be 6.85 and the solubility to be 75 μM. Thus, it seems that the p*K*_a_ was predicted to be too high, while the solubility was predicted to be too low.

The p*K*_a_ of benzoic acid, as listed in PubChem is 4.19. Our model shows that the intrinsic p*K*_a_ should be equal to 3.92. The value in the literature appears to be enlarged by approximately 0.3 pH units. However, the p*K*_a_ of benzydamine is listed in PubChem as 9.27, while our model shows that the intrinsic p*K*_a_ should be equal to 9.60. The value in the literature appears to be diminished by 0.3 pH units. Different directions for benzoic acid and benzydamine are due to different protonation charges of the molecule. Negatively charged compounds shift the p*K*_a_ upward, while the positively charged ones shift it downward. If the concentration used for p*K*_a_ determination is approximately equal to the solubility of the compound, then the p*K*_a_ will be shifted approximately by 0.3 pH units from the intrinsic p*K*_a_.

So we see that the literature tends to be affected by the p*K*_a_ shift caused by aggregation. The values were determined at some practical concentrations where some invisible aggregate was present that affected the p*K*_a_ measurements. Therefore, it would be beneficial to consider all equilibria, including the deprotonation and dissolution–aggregation, in the determinations of any compound’s intrinsic p*K*_a_ and intrinsic solubility.

## Conclusion

This study investigated the relationship between aqueous solubility and the observed p*K*_a_ of charged compounds, identifying a dependence between them. It was proposed that in water, the compound is found to exist in three forms: dissolved-charged, dissolved-uncharged, and aggregated-uncharged. The observed p*K*_a_ was dependent on the total concentration of the compound and the ‘active concentration of the aggregated form,’ whereas the intrinsic p*K*_a_ and intrinsic solubility were independent of both total added concentration and pH. The findings align with the proposed model’s framework, emphasizing the role of active concentration in determining the compound’s behavior.
